# London Hospitals without Medical Schools

**Published:** 1919-12-20

**Authors:** 


(*a*) Immediate Needs.

£25,000 to repay loan to bank. £20,000, approximate deficit in income during 1919.

(*b*) Needs for 1920.

£20,000 to meet the estimated deficit during 1920.

(*Signed*) ALFRED LANGTON, *Chairman.*

### ST. BARTHOLOMEW'S HOSPITAL.

(*a*) Immediate Needs.

Funds for the Nurses' Home rebuilding, £150,000.

(*b*) Needs for 1920.

### LONDON HOSPITALS WITHOUT MEDICAL SCHOOLS.

### BOLINGBROKE HOSPITAL.

(*a*) Immediate Needs.

To wipe off loan by bankers to about £9,000.

(*b*) Needs for 1920.

Increase of donations and subscriptions to carry on work of hospital efficiently.

(*Signed*) E. G. THORNE, *Vice-Chairman.*

### HAMPSTEAD GENERAL AND NORTH-WEST LONDON HOSPITAL.

(*a*) Immediate Needs.

£5,000 to extinguish debt and for repairs and improvements.

(*b*) Needs for 1920.

£10,000 to assist in meeting current expenses, improvements, and repairs.

(*Signed*) ALFRED LANGTON, *Chairman.*

### KENSINGTON AND FULHAM GENERAL HOSPITAL.

(*a*) Immediate Needs.

Funds to purchase food and drugs are urgently required.

(*b*) Needs for 1920.

Many more subscriptions and donations are required owing to great increase in cost of everything, especially food, drugs, and labour, otherwise 1920 will see the closing down of some of the beds.

(*Signed*) H. A. GODSON BOHN, *Chairman.*

### ITALIAN HOSPITAL, Queen Square, W.C. 1.

(*a*) Immediate Needs.

Special contributions to enable the hospital to end the year without a deficit.

(*b*) Needs for 1920.

Additional support, more especially in the form of new annual subscribers.

(*Signed*) STUART COATS [Sir], *Chairman.*

### LONDON HOMŒOPATHIC HOSPITAL.

(*a*) Immediate Needs.

We have an overdraft at the bank of £11,000, which must be paid off.

(*b*) Needs for 1920.

An increased annual income of £10,000. Help by legacy, donation, and annual subscriptions.

(*Signed*) R. H. CAIRD, J.P., *Chairman.*

### METROPOLITAN HOSPITAL, Kingsland Road, London, E. 8.

(*a*) Immediate Needs.

(1) £18,000 for debts; new annual subscriptions, £10,000; £10,000 mortgage on freehold site (income, assured, under £700). (2) Sympathy and help from everyone, and encouragement in big effort being put out.

(*b*) Needs for 1920.

£16,000 for maintenance. Help for (*a*) improved accommodation for nurses; (*b*) more space, for all departments crowded; (*c*) better casualty and pathological departments.

(*Signed*) A. LISTER HARRISON, *Acting Chairman.*

### MILLER GENERAL HOSPITAL FOR SOUTH-EAST LONDON.

(*a*) Immediate Needs.

At least £4,000 to wipe out deficit on the year's working.

(*b*) Needs for 1920.

£18,000 for maintenance. £175,000 for the much-overdue extension.

(*Signed*) JOHN H. ROBINSON, *Chairman.*

### ROYAL NATIONAL ORTHOPÆDIC HOSPITAL.

(*a*) Immediate Needs.

£20,000 yearly, in voluntary contributions, for maintenance alone.

(*b*) Needs for 1920.

£100,000 for extension of present buildings, and a sum for a country branch hospital of 200 beds.

(*Signed*) ARTHUR MORLEY, *Secretary* (for Chairman).

### POPLAR HOSPITAL FOR ACCIDENTS.

(*a*) Immediate Needs.

£5,000 for general repairs of the hospital, annual subscriptions, and donations.

(*b*) Needs for 1920.

£50,000 for improvements in casualty department, ambulance arrangements, *x*-ray operating-room, ditto, for O.P.s, more sleeping accommodation for nurses, a new ward for children.

(*Signed*) PERCY ROGERS, B.A.

### WEST LONDON HOSPITAL, Hammersmith, W. 6.

(*a*) Immediate Needs.

£25,000 to pay off debt.

(*b*) Needs for 1920.

£100,000 for extension purposes (new out-patient department and new wards to accommodate 150 beds). This extension is urgently necessary to cope with the work.

(*Signed*) GEORGE F. MARSHALL, *Chairman.*

